# Stretching of *cis*-formic acid: warm-up and cool-down as molecular work-out

**DOI:** 10.1039/c9sc01555h

**Published:** 2019-05-16

**Authors:** Katharina A. E. Meyer, Martin A. Suhm

**Affiliations:** a Institut für Physikalische Chemie , Georg-August-Universität Göttingen , Tammannstr. 6 , 37077 Göttingen , Germany . Email: msuhm@gwdg.de ; Fax: +49 551 39 33117 ; Tel: +49 55139 33111

## Abstract

A new technique to rotationally simplify and Raman-probe conformationally and vibrationally excited small molecules is applied to the *cis*–*trans* isomerism of formic acid. It quintuples the previously available gas phase vibrational data base on this excited form of a strongly anharmonic planar molecule despite its limited spectral resolution. The newly determined *cis*-formic acid fundamentals allow for a balanced vibrational benchmark on both rotamers of formic acid. Assuming the adequacy of vibrational perturbation theory, it reveals weaknesses of standard methods for these systems like B3LYP-D3(BJ)/aVQZ VPT2 or PBE0-D3(BJ)/aVQZ VPT2. The functionals ωB97-XD and M06-2X additionally suffer from severe integration grid size and symmetry dependencies. The vibrational benchmark suggests B2PLYP-D3(BJ)/aVQZ VPT2 and MP2/aVQZ VPT2 as partially competitive and in any case efficient alternatives to computationally demanding coupled cluster vibrational configuration interaction calculations. Whether this is due to fortuitous compensation between electronic structure and vibrational perturbation error remains to be explored.

## Abstract 2

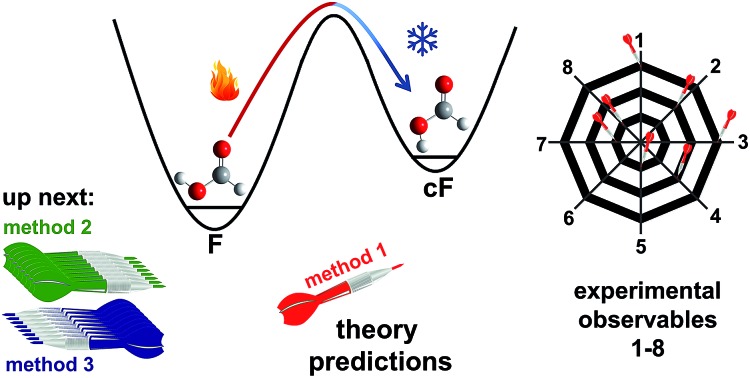
The spectroscopic data base for *cis*-formic acid is considerably extended to make it fit for experimental benchmarking of vibrational calculation tools.

## 1 Introduction

Vibrational spectra of small molecules effectively probe the quality of potential energy hypersurface (PES) predictions, when combined with accurate anharmonic calculations.^1^ Typically, an intense interplay between theory and experiment initially converges the performance for a set of low quantum number states around the global minimum. To explore the globality of a PES, it is then desirable to add experimental data on a secondary minimum structure. Its quantum states start locally, but evolve into mixed structure states at higher excitation, probing the transition state region as well. For three atoms, HCN/HNC is the paradigmatic example.^2^ For four atoms, the simultaneous description of the nearly isoenergetic formaldehyde molecule and H_2_–CO complex is challenging.^3^,^4^ For five atoms, the *cis*–*trans* isomerism of formic acid is arguably one of the most interesting systems, calling for suitable experimental reference data for the higher-energy *cis*-form. These have been surprisingly scarce until very recently, with a single exception.^5^

As the smallest carboxylic acid, the formic acid monomer has been addressed by a plethora of theoretical^6^–^15^ and experimental studies.^5^,^16^–^55^ The isomerisation from the ground state *trans*-form to the higher-energy *cis*-form has been of particular interest,^5^,^14^,^22^,^41^,^42^ even when looking at processes in the interstellar medium.^56^ When it comes to the vibrations of *cis*-formic acid, matrix isolation has been the method of choice thus far, because the possibility of long irradiation times allows for a significant formation *via* laser excitation of the *trans*-form.^35^,^41^,^42^,^49^,^50^,^57^ Since the matrix environment shifts the band positions compared to the gas phase, a direct comparison with predicted band positions of modern quantum chemical methods would require a very challenging description of the environment. Accurate theoretical predictions for the isolated *cis*-isomer thus suffer from a lack of gas phase experimental reference data. Two recent studies where this applies are by Tew and Mizukami^14^ from 2016 and by Richter and Carbonnière^15^ from 2018.

Due to the fairly large energy difference of 1365 ± 30 cm^–1^ between both rotamers of formic acid,^22^ vibrational gas phase data on the *cis*-form are rare. The first gas phase band position of *cis*-formic acid has been published in 2006 by Baskakov and co-workers, who studied the out-of-plane bending vibration with high resolution FTIR spectroscopy.^5^ Only very recently, it was complemented by a second example obtained as a side effect when studying excitonic C=O stretch coupling in jet-cooled carboxylic acid dimers.^54^ This observation has triggered the present work, which represents a systematic study of all four valence stretching modes of *cis*-formic acid. It is based on a powerful new Raman scattering approach of thermally populated and rapidly re-cooled molecules. Instead of conserving the conformational excitation by cryogenic matrix trapping,^58^ the spectra are rotationally simplified by supersonic expansion. Vibrational and high barrier conformational excitation is largely trapped and can be probed without environmental distortion as a function of initial gas temperature. Back-tunnelling to the *trans*-form is also not an issue on the time scale of the jet expansion, making it an “easy” experiment.^59^ By a 400% increase of perturbation-free *cis*-formic acid vibrational frequencies after a decade of stagnation, we provide the first systematic access to the performance of quantum chemical methods towards this model system. This decreases the likelihood of accidental error compensation between electronic structure, vibrational treatment, and matrix shifts for *cis*-formic acid by orders of magnitude.

The structure of this paper is as follows: we briefly illustrate the general approach of how the spectra of *cis*-formic acid were recorded, followed by a detailed analysis of the spectra and a first benchmark of vibrational perturbation theory and literature variational data against the experimental data. It is hoped that this progress will trigger further growth in the experimental data base and its use in benchmarking the global PES of formic acid and pentatomic vibrational treatments.

## 2 Methods

### 2.1 Experimental

A detailed description of the experimental set-up can be found in previous publications.^54^,^60^ Formic acid (Acros Organics, 98+%) was seeded at 0.2% in helium and expanded through a vertical slit nozzle at 1.0 bar into the evacuated jet chamber (background pressure < 0.1 mbar). The expansion was probed by a 532 nm, 25 W continuous-wave laser from below. The scattered radiation was detected perpendicularly with respect to both the expanding flow and the laser with a monochromator equipped with a charge-coupled device camera. *cis*-Formic acid was formed in small quantities from the *trans*-rotamer by heating the nozzle and its feed line to temperatures between 100–190 °C.

### 2.2 Computations

The quantum chemical calculations shown in this work were performed with the Gaussian 09 program package (revision E.01)^61^ using a pruned ultra fine integration grid (99 590). This grid is finer than the default of Gaussian 09 (fine grid, (75 302)).^61^ The employed methods are B3LYP,^62^,^63^ B2PLYP,^64^ MP2,^65^,^66^ M06-2X,^67^ ωB97-XD,^68^ PBE0,^69^ and PM3.^70^,^71^ Grimme's pairwise dispersion correction (D3) in combination with Becke–Johnson (BJ) damping has been added for B3LYP, B2PLYP, and PBE0.^72^ For all methods, an augmented quadruple-zeta (aVQZ) basis set has been chosen. Additional augmented double-(aVDZ) and triple-zeta (aVTZ) calculations were carried out for MP2 and B2PLYP-D3(BJ). Moreover, the production calculations have been performed without the use of symmetry, employing opt = tight convergence.

The assignment of *cis*-formic acid fundamentals has been supported by scaled, harmonic frequency calculations at the B3LYP-D3(BJ)/aVTZ level, which have proven to yield sufficient agreement in a previous study.^54^ For the vibrational benchmark in Section 3.2, anharmonic frequency calculations were performed at all levels listed above using vibrational perturbation theory (VPT2),^73^ as implemented in Gaussian 09.^61^ VPT2 was used under the default settings where resonances identified in a pre-screening are removed and treated variationally.

Additionally, exploratory VPT2 calculations utilising the *C*_s_ symmetry as well as a finer integration grid (pruned super fine integration grid (150 974)^61^) were carried out in selected cases to probe their impact on the results. A brief discussion can be found in Section 3.3.

## 3 Results and discussion

### 3.1 The stretching vibrations of *cis*-formic acid

**Fig. 1:**
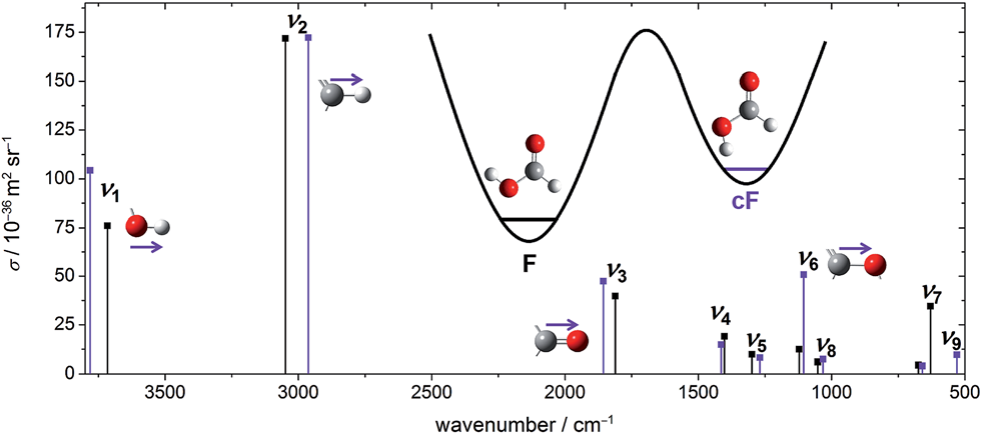
Harmonically predicted band positions and Raman scattering cross-sections *σ* (ref. 74) of all nine fundamentals of *cis*- (violet) and *trans*-formic acid (black), calculated at the B3LYP-D3(BJ)/aVTZ level.

To choose suitable spectral regions for *cis*-rotamer detection, the band positions and Raman scattering cross-sections have been predicted using B3LYP-D3(BJ)/aVTZ alongside those of the *trans*-form. The results are displayed in Fig. 1. The vibrations have been labelled according to the Herzberg nomenclature. The *cis*-formic acid vibrations with the largest scattering cross-sections are *ν*_1_, *ν*_2_, *ν*_3_, and *ν*_6_, namely the O–H, the C–H, the C=O, and the C–O stretching vibration. In fact, *ν*_6_ is the only stretching vibration with a distinctly larger scattering cross-section compared to *trans*-formic acid.

**Fig. 2:**
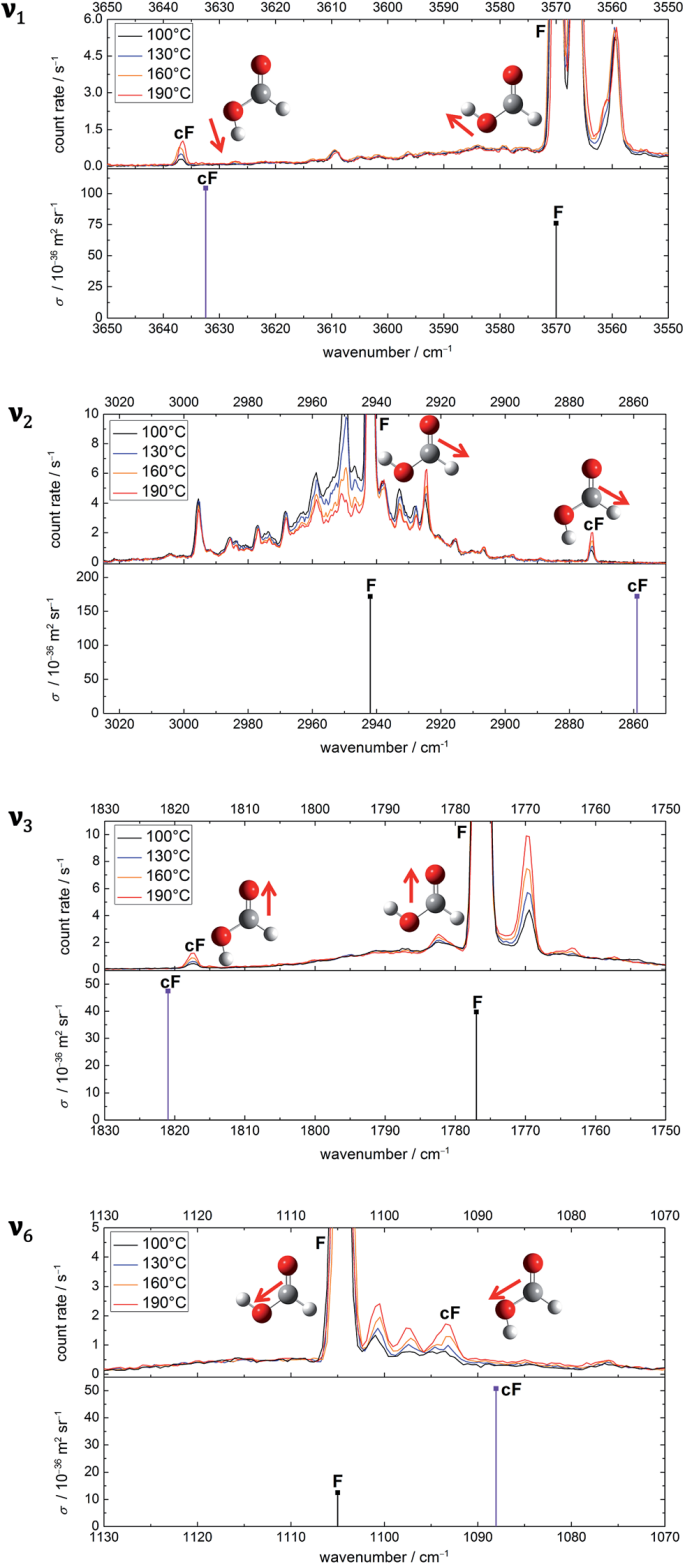
Raman jet spectra of formic acid (≈0.2%) in helium recorded at a reservoir pressure of 1.0 bar with increasing nozzle temperatures of 100–190 °C. Four spectral regions are covered at four temperatures with data acquisition times between 28 and 63 minutes. Within a spectral region, the spectra have been intensity-scaled to the *trans*-formic acid monomer band F with the lowest concentration amongst the four temperatures. Below each temperature series, calculated, *v*_*i*_(F)-scaled harmonic band positions (B3LYP-D3(BJ)/aVTZ level) of cF and F are shown.

The experimental spectra of these four vibrational modes of both rotamers (cF, F) can be found in Fig. 2 alongside harmonic, individually F-scaled B3LYP-D3(BJ)/aVTZ calculations below the spectra. For each spectral region, four spectra with increasing nozzle temperature have been recorded. These temperature series have been intensity-scaled to the *trans*-monomer band of lowest intensity amongst the four. Consequently, any hot band, *i.e.*, *cis*-formic acid or a non-isomeric hot band originating from thermally populated low-lying energy levels of *trans*-formic acid, should increase in intensity with nozzle temperature, whereas any formic acid cluster band will decrease due to thermal dissociation.

The spectra in the O–H stretching region show one band that increases in intensity with temperature at 3637 cm^–1^. The band position is in good agreement with the harmonically calculated, *ν*_1_(F)-scaled band position of cF with a deviation of only 5 cm^–1^. Either the anharmonicity of F and cF is similar or there is error compensation with the density functional used. Another way of validating this assignment is to compare the intensity ratio of the cF (3637 cm^–1^) and F (3570 cm^–1^) bands with the energy difference of both forms. The harmonically calculated energy difference of 15.9 kJ mol^–1^ (B3LYP-D3(BJ)/aVTZ with zero point energy correction) is just below the error bounds of the only experimental value of 1365 ± 30 cm^–1^ by W. Hocking.^22^ Neglecting differences in the partition function of the two complexes, this corresponds to a population of 1–2% of *cis*-formic acid at 190 °C. After correction by the theoretical cross-section ratio, the ratio of the experimental band integrals provides a *cis*-abundance of 2%, thus reaffirming the cF assignment. The additional bands downshifted compared to the O–H stretching vibration of *trans*-formic acid at 3560 cm^–1^ and 3566 cm^–1^ are most likely *trans*-formic acid combination bands of *ν*_2_ with the lowest frequency vibrations *ν*_7_ (3560 cm^–1^) and *ν*_9_ (3566 cm^–1^), which benefit from the large Raman scattering cross-section of the C–H stretching vibration. The former is in good agreement with the predicted values of Tew and Mizukami (3566 cm^–1^)^14^ as well as Richter and Carbonnière (3558 cm^–1^)^15^ and the latter with a prediction of Tew and Mizukami,^14^ who reported (*ν*_2_ + *ν*_9_) in Fermi resonance with (*ν*_3_ + 3*ν*_9_) at 3571 cm^–1^ and 3579 cm^–1^.

The *ν*_2_ region is spectrally more congested due to its low sensitivity to hydrogen bonding. In the spectral windows 2970–2945 cm^–1^ and 2935–2925 cm^–1^, there are several bands that decrease in intensity with temperature, *i.e.*, cluster bands. The broad underlying signal is due to rovibrational O and S branches of *ν*_2_. As opposed to the O–H stretching region, there are two distinct bands increasing in intensity with temperature at 2925 cm^–1^ and 2873 cm^–1^. The latter deviates from the predicted band position of *cis*-formic acid by 14 cm^–1^. The amount of *cis*-formic acid at 190 °C deduced from the integrated intensities of the bands amounts to 1%, which fits the energy difference, as detailed above. Therefore, the band at 2873 cm^–1^ can be assigned to cF. The second hot band at 2925 cm^–1^ is shifted by –17 cm^–1^ from the fundamental of F (2942 cm^–1^). For an assignment to F, two things need to be considered: firstly, the shift directly yields the off-diagonal anharmonicity constant *x*_2*i*_ between *ν*_2_ and a low-lying energy level *v*_*i*_ that is thermally populated. Secondly, the intensity ratio is dependent on the Boltzmann population of that level and yields the excitation energy of the latter. Hence, the assignment can be checked by comparing the experimentally determined anharmonicity constant and intensity with the calculated values for the lowest-lying energy levels of *trans*-formic acid. From the anharmonicity matrix elements in Table 1 it is apparent, that the hot band originating from *ν*_7_ will most likely overlap with the fundamental, whereas the hot band originating from *ν*_9_ (and *ν*_6_) could overlap with a cluster band at 2938 cm^–1^ causing the highest nozzle temperature spectrum (red) and the lowest nozzle temperature spectrum (black) to have similar intensities. However, due to the spectral congestion in this area, reliable assignments are not feasible. Additional depolarisation measurements to subtract the O and S branches from the sharp Q peak are currently ongoing and will be addressed in detail in a subsequent publication. Here we focus on the straightforward assignment of the 2925 cm^–1^ band. Its observed shift of –17 cm^–1^ perfectly matches the calculated anharmonicity constant *x*_28_. The expected intensity ratio at 190 °C of around 4% approaches the observed ratio of 3%, so that it can be assigned to *ν*_2_ + *ν*_8_ – *ν*_8_.

**Table 1:** Calculated anharmonic (VPT2) band positions (in cm^–1^) of *trans*-formic acid alongside calculated diagonal (italics) and off-diagonal anharmonicity constants *x*_2*i*_, *x*_3*i*_, and *x*_6*i*_ (in cm^–1^) of *ν*_2_, *ν*_3_, and *ν*_6_ with all nine fundamentals

*v*_*i*_	Band position	B3LYP-D3(BJ)/aVTZ VPT2		
		*x*_2*i*_	*x*_3*i*_	*x*_6*i*_
1	3533	–3.5	–1.1	–0.9
2	2892	–61.9	–13.4	–2.3
3	1779	–13.4	–9.3	–4.1
4	1374	–20.4	–0.3	–6.2
5	1219	–6.1	+2.8	–14.3
6	1089	–2.3	–4.1	–6.2
7	622	+0.4	–6.2	–3.7
8	1031	–17.1	–5.4	–3.7
9	643	–2.8	–0.4	–5.6

A first analysis of the *ν*_3_ spectral region at nozzle temperatures of 23 °C, 110 °C, 140 °C, and 170 °C can be found in a previous publication.^54^ Omitting the room temperature measurement in this work is intentional, as it is heavily congested with cluster bands due to the fairly high concentrations and reservoir pressures chosen. These, however, are essential to obtain high monomer signals of F, and especially cF, after thermal dissociation of the clusters. Due to an improved signal-to-noise ratio compared to the previous measurements as well as the somewhat higher upper nozzle temperature available in the present work, we have reanalysed the *ν*_3_ region. Briefly, the *cis*-formic acid band can be seen at 1818 cm^–1^, which deviates from the calculated, *ν*_3_(F)-scaled band position by 3 cm^–1^. The hot band downshifted by 7 cm^–1^ from F can most likely be attributed to *ν*_3_ + *ν*_7_ – *ν*_7_, with a negligible (1 cm^–1^) deviation of the calculated anharmonicity constant *x*_37_ compared to the experimentally observed value and a reasonable Boltzmann population match (14% from the level energy and 10% from the Raman spectrum). The hot band intensity qualitatively rules out major contributions from higher energy levels such as *ν*_8_. There are two weaker potential hot bands shifted from F by +6 cm^–1^ and –13 cm^–1^ with intensities of around 1–2% compared to F. An assignment is not possible since the shifts do not match the predicted anharmonicity constants (*cf. x*_39_, *x*_38_, *x*_36_, and *x*_35_ in Table 1). As previously seen, the intensity ratio gives only a rough estimate of the energy level and as such, cannot serve as a stand-alone assignment criterion. Overall, this highlights the importance of the *ν*_2_ region with its much higher monomer signal due to the large Raman scattering cross-section of the C–H compared to the C=O stretching vibration.

In the C–O stretching region, three hot bands can be seen downshifted from the fundamental *ν*_6_ of *trans*-formic acid at 1105 cm^–1^. The shifts amount to –4 cm^–1^, –7 cm^–1^, and –11 cm^–1^ with intensities of around 7%, 3%, and 7% of *ν*_6_ at 190 °C. To assign *ν*_6_ of *cis*-formic acid, the shifts are compared to the calculated anharmonicity constants *x*_6*i*_ in Table 1. The predicted anharmonicity constants *x*_67_ and *x*_68_ agree (–3.7 cm^–1^). In addition, *x*_69_ and *x*_66_ are very similar (–5.6 cm^–1^ and –6.2 cm^–1^). Therefore, it seems likely that the bands at 1101 cm^–1^ and 1097 cm^–1^ are a result of overlapping hot bands. The slightly higher intensity of the former is a result of the greater overlap with the fundamental and the lower energy of *ν*_7_ and *ν*_8_ compared to *ν*_9_ and *ν*_6_. The next higher energy level is *ν*_5_ with a predicted band position of 1219 cm^–1^. A hot band originating from *ν*_5_ is expected to be shifted by –14.3 cm^–1^ from *ν*_6_ of *trans*-formic acid (*cf.*Table 1), which is close to the experimentally observed shift of –11 cm^–1^ of the third hot band. However, the intensity of that band is with 7% of *ν*_6_ distinctly larger than the expected 2% from thermal population at 190 °C, especially considering that the observed intensities of all other hot bands are smaller than or equal to the predicted values. Hot bands from higher energy levels can therefore also be ruled out as these should have even lower intensities. The predicted band position of *cis*-formic acid deviates by –5 cm^–1^ from the band at 1093 cm^–1^, which falls within the accuracy of the *ν*_6_(F)-scaled harmonic B3LYP-D3(BJ)/aVTZ calculations. Additionally, the observed intensity matches the calculated energy difference between both rotamers, considering the four times larger predicted scattering cross-section of *ν*_6_ of *cis*-formic acid compared to the *trans*-form (*cf.*Fig. 2). Consequently, the band at 1093 cm^–1^ can be predominantly assigned to the C–O stretching vibration of *cis*-formic acid.

The band positions of all stretching vibrations of *cis*-formic acid as well as that of the out-of-plane O–H bending vibration (*ν*_9_) determined from high resolution FTIR measurements^5^ are summarised in Table 2 in comparison to the values obtained in an argon matrix by Maçôas and co-workers.^42^ The argon matrix shifts range from +27 to –20 cm^–1^ or +23 to –21 cm^–1^, dependent on the matrix site. This scatter is of a similar order of magnitude as the *cis*–*trans* spectral differences themselves, which are also listed in Table 2. It is therefore evident that band positions in a perturbation-free environment are crucial for a direct comparison with theory values such as those of Tew and Mizukami^14^ and Richter and Carbonnière.^15^

**Table 2:** Band positions of the fundamentals *v*_*i*_ of *cis*-formic acid (in cm^–1^) obtained in a supersonic jet expansion probed with Raman spectroscopy (this work) compared to literature values

*v*_*i*_	Jet (this work)	Gas phase^5^	Shift^*a*^ (cF–F)	Ar matrix^*b*^ ^,^^42^	Matrix shift^*c*^
1	3637		+67	3617.2/3615.9	–21/–21
2	2873		–69	2899.5/2896.3	+27/+23
3	1818		+41	1808.0/1806.9	–10/–11
4				/1391.8	
5				1243.4/1248.9	
6	1093		–11	1107.3/1104.6	+14/+12
7				662.3/662.3	
8					
9		493.420509(7)	–147.30467(1)	502.9/505.3	–9.5/–11.9

^*a*^gas phase band position shifts from the *trans*- to the *cis*-rotamer.

^*b*^site 1/site 2 are two dominant trapping sites.

^*c*^band position shift of the values of both matrix sites compared to the gas phase.

### 3.2 Vibrational benchmark

So far, the band positions of *cis*-formic acid have been compared to *v*_*i*_(F)-scaled harmonic band positions calculated at the B3LYP-D3(BJ)/aVTZ level, which has shown to be quite valuable in supporting the assignment. The small size of the formic acid monomer and its structural rigidity enable anharmonic vibrational perturbation theory calculations (VPT2),^73^ which have proven to be robust for the *trans*-formic acid monomer at various levels of theory in a study of the *trans*-formic and -acetic acid monomers and their nitrogen clusters.^53^ This is less the case for the *trans*-acetic acid monomer, where the presence of the floppy methyl groups resulted in instabilities such as a wavenumber increase of the lowest frequency vibration compared to the harmonic case. The newly determined band positions of *cis*-formic acid thus enable a significantly extended VPT2 benchmark involving both rotamers, which should not suffer from such methyl torsion instabilities.

**Fig. 3:**
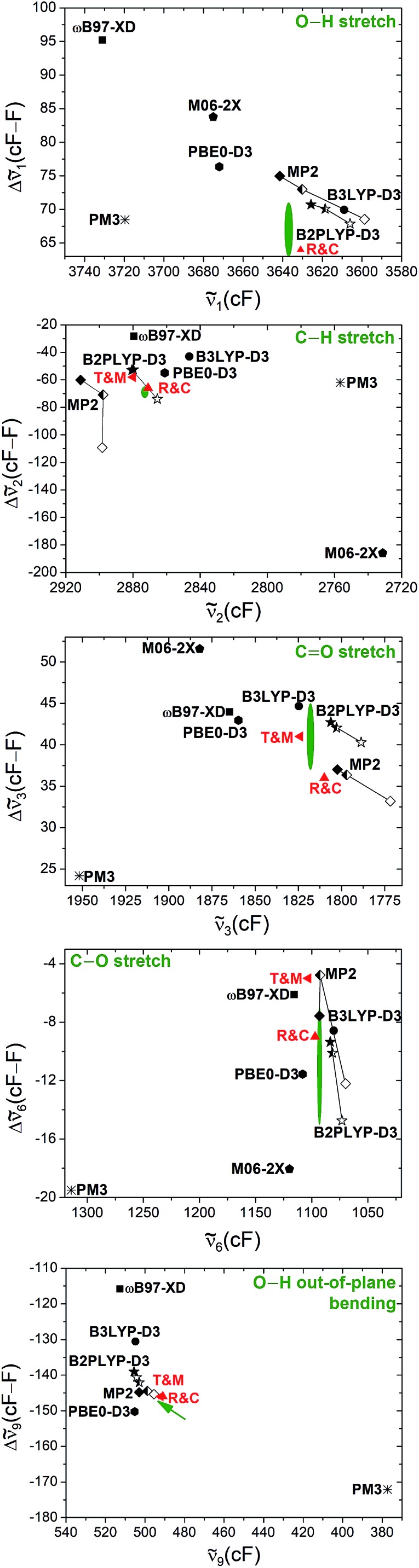
Anharmonic (VPT2), theoretical prediction of the shift between the band positions of the *cis*- and *trans*-rotamers of formic acid for five fundamentals (*ν*_1_, *ν*_2_, *ν*_3_, *ν*_6_, and *ν*_9_) plotted against the absolute band positions of the *cis*-form. The basis set size is encoded in the filling of the symbols. All black, filled symbols have been calculated with an aVQZ basis set. For the half-empty and the empty symbols, smaller basis sets have been used, namely aVTZ and aVDZ. The red triangles represent the band positions and shifts obtained from VCI calculations of Tew and Mizukami^14^ (T & M) and MCTDH calculations of Richter and Carbonnière^15^ (R & C). The green ellipsis shows agreement with experiment, whereby the size indicates the conservatively estimated experimental accuracy of ±2 cm^–1^ for *ν̃*_*i*_(cF) and ±4 cm^–1^ for Δ*ν̃*_*i*_(cF–F). The experimental band positions of the stretching vibrations (*ν*_1_, *ν*_2_, *ν*_3_, *ν*_6_) have been obtained in this work and the values of the torsional modes *ν*_9_ of both rotamers (tip of the arrow) have been taken from ref. 5 and 44. Further details can be found in the text.

The experimental values that will be employed in the benchmark are the five band positions of *cis*-formic acid as well as the band position difference between the *cis*- and *trans*-fundamentals. The methods tested are the same as in ref. 53, namely B3LYP-D3(BJ), B2PLYP-D3(BJ), MP2, PBE0-D3(BJ), ωB97-XD, M06-2X, and in addition also PM3, all as implemented in Gaussian 09.^61^ For all methods, an augmented quadruple-zeta (aVQZ) basis set has been used. Additional augmented double-zeta (aVDZ) and triple-zeta (aVTZ) calculations have been performed for MP2 and B2PLYP-D3(BJ). The benchmarking plots can be found in Fig. 3. The accuracy of the band positions with the Raman set-up used in this work is about 1 cm^–1^ (ref. 60) and the full width at half maximum of all bands is around 2 cm^–1^, leading to a conservative error estimate of ±2 cm^–1^ for the band positions and twice the amount for the shift. The green ellipsis in each plot thus shows the area that is in acceptable agreement with experiment. Since *ν*_9_ has been measured with high resolution FTIR spectroscopy with a precision on the order of ∼10^–5^ cm^–1^ and a somewhat lower accuracy,^5^,^44^ a green arrow points towards the exact band position and shift in the bottom panel of Fig. 3. Additionally, the results of Tew and Mizukami (T & M)^14^ and Richter and Carbonnière (R & C)^15^ have been included for all vibrations. Briefly, Tew and Mizukami have fitted a potential energy surface based on 17 076 energies calculated at the CCSD(T)(F12*)/cc-pVTZ-F12 level. The vibrational levels were obtained by using vibrational configuration interaction (VCI) with an internal coordinate path Hamiltonian for the isomerisation path connecting both rotamers.^14^ Richter and Carbonnière have constructed a valence coordinate potential energy surface at the CCSD(T)-F12a/aug-cc-pVTZ level and carried out the vibrational energy calculations with the improved relaxation multi-configuration time-dependent Hartree (MCTDH) method.^15^

Firstly, the performance of VPT2 calculations at various levels of theory will be discussed before these will be compared with the VCI and MCTDH calculations. One should note that for the O–H stretching vibration this comparison can solely be made with the MCTDH calculations, as the theoretical band position of the *cis* O–H stretching vibration has not been reported by Tew and Mizukami. Due to error compensation, a better agreement between experiment and the tested methods is typically achieved for the shift between the *cis*- and *trans*-rotamers. The absolute band position is predicted correctly in two cases, namely with MP2/aVTZ and MP2/aVQZ for the C–O stretching vibration (*ν*_6_). All other methods fail to predict the *cis*-formic acid band positions correctly despite generous experimental error bars for the stretching vibrations. An accurate prediction of *ν*_9_ (and the respective shift) is evidently unrealistic due to the high accuracy of the high resolution measurements. The lower resolution Raman spectra are seen to be fully adequate to challenge theory on an absolute wavenumber scale. The vibrations where the shift is predicted within the experimental error for most methods are the C=O and C–O stretching vibrations, whereas the largest divergence is observed for the C–H stretching vibration. This is not surprising as the C–H stretching vibration is prone to stretch-bend Fermi resonance, although the VPT2 code employed^61^ attempts to include such pronounced resonances. Consequently, part of the discrepancy may be due to a poor vibrational description by VPT2 rather than the electronic structure calculation. The particularly drastic failure for M06-2X is caused by an inversion of the predicted energy sequence for the C–H stretch fundamental and C–H bending overtone of *cis*-formic acid, which is amplified by Fermi resonance. If the band labels are switched, the agreement increases significantly – the severe underestimation of the band position of –142 cm^–1^ (Fig. 3) changes to an overestimation of +10 cm^–1^. The band position shift improves from –186 cm^–1^ (Fig. 3) to –34 cm^–1^, compared to the experimental value of –69 cm^–1^.

A comparison of the vibrationally averaged, calculated rotational constants for all methods with the experimental values for *cis*-formic acid obtained by Winnewisser and co-workers^39^ is shown in Table 3. Small individual deviations on the order of ±0.5% are observed for B2PLYP-D3(BJ), B3LYP-D3(BJ), and MP2, larger deviations of up to 1–2% for M06-2X, ωB97-XD, and PBE0-D3(BJ), and very large deviations for PM3. The average deviation over all three rotational constants (last row in Table 3) supports the overall agreement with the experimental structure. The B3LYP-D3(BJ) structure shows the best agreement with a divergence of –0.1%, directly followed by B2PLYP-D3(BJ) (–0.3%). For MP2, the divergence is slightly larger as all rotational constants are underestimated and thus, do not compensate each other. The same is valid for M06-2X, ωB97D, and PBE0-D3(BJ), where all constants are overestimated.

**Table 3:** Relative deviations (in %) of VPT2 (aVQZ) rotational constants of *cis*-formic acid from the experimental values of Winnewisser and co-workers^39^

	B2PLYP-D3(BJ)	B3LYP-D3(BJ)	MP2	M06-2X	ωB97-XD	PBE0-D3(BJ)	PM3
Δ*A*_0_/*A*_0_	+0.1	+0.4	–0.3	+1.4	+1.2	+1.1	–18.9
Δ*B*_0_/*B*_0_	–0.5	–0.3	–0.5	+0.6	+0.6	+0.6	+9.8
Δ*C*_0_/*C*_0_	–0.4	–0.3	–0.5	+0.7	+0.6	+0.6	+5.3
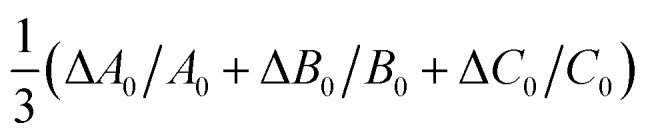	–0.3	–0.1	–0.4	+0.9	+0.8	+0.8	–1.3

A comparison of the individual performance of the methods for the determination of *ν̃*_*i*_(cF) and Δ*ν̃*_*i*_(cF–F) clearly illustrates that there are few reliable methods. In case of PM3, this is not surprising. It is the only method that fails to predict the energetic order of the vibrations correctly with *ν*_4_ and *ν*_6_ switched. Other methods with particularly severe deviations from experiment are ωB97-XD (*cf. ν*_1_ and *ν*_9_) and M06-2X (*ν*_1_, *ν*_2_, and *ν*_9_). The large underestimation of *ν̃*_2_(cF) and Δ*ν̃*_2_(cF–F) of M06-2X is enhanced by a level switch between resonance partners, as discussed above. All other methods predict the correct sequence of fundamental and overtone. Another numerical or fundamental deficiency of M06-2X/aVQZ VPT2 is the incorrect sign of the total anharmonicity of *ν*_9_ of *cis*-formic acid, which gives rise to a large overestimation of the anharmonic band position (+163 cm^–1^). In combination with an overestimation of the negative anharmonicity of *ν*_9_ of *trans*-formic acid, this results in a severe overestimation of the shift (+308 cm^–1^) between both rotamers. As such, this data point has been omitted from Fig. 3. The PBE0-D3 calculations match the experimental shifts in two cases (*ν*_3_ and *ν*_6_), whereas B3LYP-D3(BJ) predicts the shifts correctly in three of the five cases (*ν*_1_, *ν*_3_ and *ν*_6_). Both exhibit similar deviations with respect to the band positions. Since the rotational constant prediction of B3LYP-D3(BJ) is also more accurate, it is the overall better choice. MP2 is particularly good for the description of the lower frequency modes *ν*_6_ and *ν*_9_ and overshoots for *ν*_1_ and *ν*_2_. For *ν*_3_, an agreement with the shift is reached with the largest basis set aVQZ. It is generally rewarding that basis set sensitive methods tend to move towards the experimental region with increasing basis set size (*cf.*Fig. 3). Another reliable method is B2PLYP-D3(BJ), which predicts the shifts correctly in three cases (*ν*_1_, *ν*_3_ and *ν*_6_) and shows only small deviations for the other two. The band positions are slightly, but consistently underestimated, apart from *ν*_2_, where a small overestimation occurs for the larger basis sets, and *ν*_9_, which is slightly overestimated for all basis set sizes.

The band positions and shifts obtained from the VCI calculations of Tew and Mizukami^14^ show good agreement with experiment. For all stretching vibrations, the band positions are overestimated and *ν*_9_ differs solely by –1 cm^–1^. With regard to the shifts, only one is predicted within the experimental uncertainty (*ν*_3_), but the shift of *ν*_9_ differs solely by about 1 cm^–1^. Deviations are generally small and on the same order as for B2PLYP-D3(BJ)/aVQZ VPT2 or MP2/aVQZ VPT2. The agreement of the MCTDH calculations of Richter and Carbonnière^15^ with experiment is even slightly better. The band position shifts between both rotamers are predicted accurately for all stretching vibrations apart from *ν*_3_, where the value is with 36 cm^–1^ just outside the experimental confidence interval (41 ± 4 cm^–1^). The band position of the C–H stretching vibration is predicted within the experimental accuracy and the *ν*_9_ prediction deviates only by 2 cm^–1^. However, the latter gas phase value was the only band position of formic acid known in the gas phase before ref. 14 and 15 were published, whereas the other *cis*-values were true predictions for the isolated molecule.

**Fig. 4:**
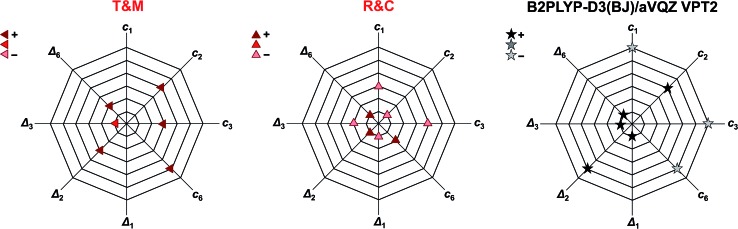
Agreement of the predicted anharmonic band positions of the stretching vibrations of *cis*-formic acid (*c*_1,2,3,6_) as well as band position shifts between *cis*- and *trans*-formic acid (*Δ*_1,2,3,6_) with experiment. The octagon size displays the deviation from experiment in units of experimental confidence interval, *i.e.*, the smallest octagon represents agreement within the error bars of ±2 cm^–1^ for *c*_*i*_ and ±4 cm^–1^ for *Δ*_*i*_ (green ellipses in Fig. 3) and the *n*th octagon agreement within *n* experimental confidence intervals. The methods tested are the VCI calculations of Tew and Mizukami (T & M),^14^ the MCTDH calculations of Richter and Carbonnière (R & C),^15^ as well as VPT2 calculations at the B2PLYP-D3(BJ)/aVQZ level. The symbols used are the same as in Fig. 3. The color shade shows whether the experimental observable is overestimated (+), underestimated (–), or met.

Another way of visualising the agreement of the theoretical predictions of Tew and Mizukami, Richter and Carbonnière, and results obtained with vibrational perturbation theory (B2PLYP-D3(BJ)/aVQZ VPT2) with experiment is shown in Fig. 4. In these three diagrams, the eight accessible deviations from experiment are plotted in units of experimental confidence interval for all four stretching vibrations in the form of octagons. Each axis connecting two vertices of the octagons corresponds to one of the four vibrations. The two directions of each axis display the two experimental observables for each vibration, namely the *cis*-formic acid band position (*c*_*i*_) and the band position shift between *cis* and *trans* (*Δ*_*i*_). The size of the deviation from experiment is encoded in the octagon size. A point on a node with the smallest octagon translates into theoretical agreement within the experimental error bars (±2 cm^–1^ for the band position and ±4 cm^–1^ for the shifts). Correspondingly, a point on a node with the *n*th octagon implies a deviation of that value by up to *n* confidence intervals from experiment. The predicted band position for the C=O stretching vibration of *cis*-formic acid by Tew and Mizukami (1824 cm^–1^)^14^ deviates by +6 cm^–1^ from the Raman jet value of 1818 cm^–1^. Considering the experimental confidence interval of ±2 cm^–1^, the prediction for *c*_3_ lies on the third octagon, or in other words, three nodes away from the origin on the *c*_3_ axis. Note that the origin in these diagrams cannot be met due to the experimental uncertainty. The sign of the deviation is illustrated by the colour shade of the symbol, whereby a dark colour shows over- and a light colour underestimation. The intermediate shade represents an indeterminate sign of the deviation, which can be seen for the shift of the C=O stretching vibration *Δ*_3_ of Tew and Mizukami. The aforementioned consistent overestimation of the VCI calculations of Tew and Mizukami (T & M) (apart from *Δ*_3_) can thus be directly seen by the otherwise dark-coloured symbols. The MCTDH method of Richter and Carbonnière falls closer to the origin and varies more in sign. Therefore, it shows superior agreement with experiment compared to the results of Tew and Mizukami. The tendency of the B2PLYP-D3(BJ)/aVQZ VPT2 calculations to underestimate the band position *c*_*i*_ as well as its ability to predict most shifts within the experimental accuracy (smallest octagon) is illustrated. Altogether, Fig. 4 nicely sums up that the MCTDH method utilised by Richter and Carbonnière offers a slightly better description of the vibrations scrutinised here. The VPT2 calculations at the B2PLYP-D3(BJ)/aVQZ level are seen to provide a less expensive alternative. This good performance of the double hybrid functional has recently been illustrated for pyruvic acid by Barone *et al.*^75^ For formic acid, there are some interesting systematic errors, which have consequences when looking at matrix isolation spectroscopy. Superficially and surprisingly, the comparison of VPT2 anharmonic data for *trans*-formic acid only improves slightly when moving from a matrix to the gas phase.^12^ This is largely due to substantial downshifts of polar (O–H, C=O) stretching vibrations in an Ar matrix, which mimic the underestimation of these vibrations by the B2PLYP functional in the gas phase. Such good agreements for the wrong reason must be avoided in proper benchmarking. Only the gas phase comparison can provide a realistic picture of the electronic structure performance.

With regard to the previous assignment of hot bands of *trans*-formic acid, the coupling constants to levels with significant thermal population at 190 °C predicted with B3LYP-D3(BJ)/aVTZ (see Table 1) are in good agreement with those at the B2PLYP-D3(BJ)/aVQZ VPT2 level. The largest discrepancy amounts to 0.6 cm^–1^ (*x*_36_), which is below the spectral resolution of the Raman experiment.

### 3.3 Instabilities of DFT functionals

As previously mentioned in Section 2.2, all production calculations have been carried out without the use of symmetry using the pruned ultra fine integration grid of Gaussian 09.^61^ To explore the influence of symmetry and grid size, additional calculations have been performed exploiting the *C*_s_ symmetry and a finer integration grid (super fine integration grid, (150 974)).^61^ For the following analysis, the five vibrations discussed in this work have been considered for both rotamers, *i.e.*, 10 values.

**Fig. 5:**
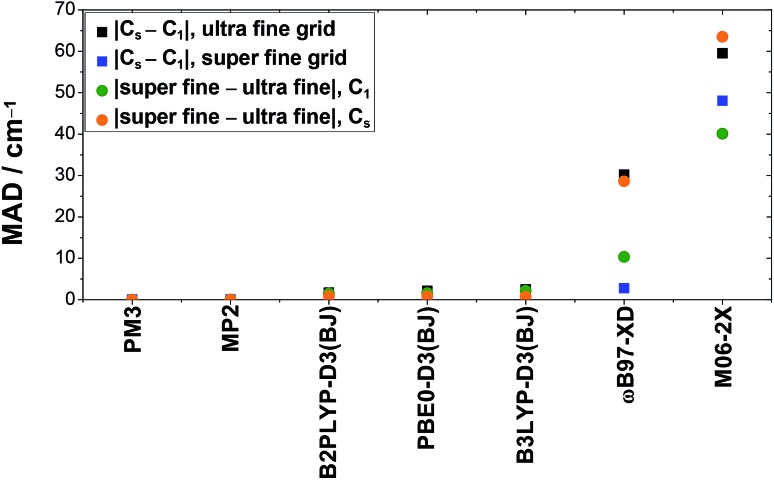
Mean absolute deviations (MAD, in cm^–1^) of anharmonically (VPT2) calculated band positions of the stretching vibrations (*ν*_1_, *ν*_2_, *ν*_3_, and *ν*_6_) and the O–H out-of-plane bending vibration (*ν*_9_) of *cis*- and *trans*-formic acid resulting from the usage of symmetry (*C*_s_) compared to no symmetry (*C*_1_) or the increase of the DFT integration grid size (super fine integration grid compared to the ultra fine integration grid), as implemented in Gaussian 09.^61^

All density functional theory methods show deviations for anharmonic frequency (VPT2) calculations with and without the use of symmetry when the integration grid size is kept constant, whereas the results obtained with PM3 and MP2 have a negligible (≤0.2 cm^–1^) dependence on symmetry. The size of the deviation depends largely on the density functional theory method used as well as on the vibration. The most sensitive vibrations of the fundamentals discussed in this work are the O–H stretching (*ν*_1_) and out-of-plane bending vibration (*ν*_9_), while the smallest deviations are observed for the C=O (*ν*_3_) and C–O stretching vibrations (*ν*_6_). For B3LYP-D3(BJ), B2PLYP-D3(BJ), and PBE0-D3(BJ), these deviations are below ±10 cm^–1^, with mean absolute deviations of 2.5 cm^–1^, 1.7 cm^–1^, and 2.1 cm^–1^ for the ultra fine integration grid, respectively. Particularly severe divergence is observed for ωB97-XD and M06-2X with discrepancies of up to –96 cm^–1^ and 133 cm^–1^, respectively. The mean absolute deviations for these methods are as large as 30.2 cm^–1^ (ωB97-XD) and 59.5 cm^–1^ (M06-2X). These can be reduced by using the finer integration grid (super fine integration grid). This is illustrated in Fig. 5, where the mean absolute deviation of the band positions using *C*_1_ and *C*_s_ symmetry is plotted for both grid sizes (black and blue squares). This decrease in divergence, however, occurs at the expense of distinctly higher computational costs. In case of ωB97-XD and M06-2X, this leads to an mean absolute deviation of 2.8 cm^–1^ and 48.0 cm^–1^. The large value for M06-2X is caused by outliers where the deviation between calculations with *C*_s_ and *C*_1_ symmetry is enhanced by using the finer grid (*ν*_9_(cF, F) and *ν*_6_(cF)).

When just the integration grid is varied and the symmetry is kept fixed (either *C*_1_ or *C*_s_), the band positions vary on average between 1–2 cm^–1^ for B3LYP-D3(BJ), B2PLYP-D3(BJ), and PBE0-D3(BJ). This is on the same order of magnitude as the symmetry effects discussed above. Again, a huge impact of the integration grid size is seen for ωB97-XD and M06-2X, where mean absolute deviations of 28.6 cm^–1^ and up to 63.5 cm^–1^ are observed (*cf.* orange and green points in Fig. 5). In both cases, the deviations are larger for the *C*_s_ symmetry, whereas for the other methods, it is the other way around.

Altogether, these symmetry and integration grid size dependent variations in anharmonic band positions of the fundamentals of *cis*- and *trans*-formic acid are on the order of magnitude of the experimental error bars for B3LYP-D3(BJ), B2PLYP-D3(BJ), and PBE0-D3(BJ). Nonetheless, one should keep in mind that individual outliers are slightly larger. Anharmonic frequency calculations with ωB97-XD and M06-2X on the other hand, show substantial differences with regard to the symmetry and integration grid chosen, so that these results must be viewed with caution, as has been discussed before.^76^,^77^ For most methods, the best agreement with experiment is achieved with the *C*_s_ symmetry and the finer integration grid. Since the improvement of the accuracy is below the experimental confidence interval for the more reliable DFT methods, if present at all, Fig. 3 and 4 would only change slightly.

## 4 Conclusions

Overall, thermal excitation combined with rapid jet quenching and Raman probing as reported in this work provides access to the four stretching vibrations of *cis*-formic acid in a perturbation-free environment. These reference data points are essential for the validation and comparison of modern quantum chemical methods towards a more global description of this model system. Recent examples are VCI calculations of Tew and Mizukami^14^ and MCTDH calculations of Richter and Carbonnière.^15^ However, it was also shown that vibrational perturbation theory can be a good compromise between accuracy and computational costs for a reasonably rigid molecule like formic acid, if combined with an adequate method for the electronic structure calculation. In this case, the double hybrid method B2PLYP-D3(BJ)/aVQZ and MP2/aVQZ offer a good compromise between accuracy and cost efficiency, in particular for differences between corresponding *cis*- and *trans*-vibrations. A benchmark examining various levels of theory revealed the failure of methods like M06-2X/aVQZ VPT2 or ωB97-XD/aVQZ VPT2 to give consistent results, partly due to numerical grid size and symmetry sensitivity. With the single gas phase value from 2006 (ref. 5) available up to a year ago, these conclusions could not have been drawn. A side effect of the thermal population of *cis*-formic acid is the significant enhancement of hot bands of *trans*-formic acid compared to room temperature spectra. The anharmonicity constants that can be deduced from these can help to validate combination band assignments, which are in some cases still under debate.^15^ Finally, further experiments such as deuteration or depolarisation experiments will help to shed more light on various debates surrounding *trans*-formic acid. A prominent example is the assignment of *ν*_5_ and the overtone 2*ν*_9_, where calculations of Tew and Mizukami^14^ and Richter and Carbonnière^15^ disagree with the experimental, infrared spectroscopic assignments of Freytes and co-workers^37^ as well as Raman spectra of Bertie and Michaelian.^27^ Additional Raman data recorded in the fashion shown here, *i.e.*, in combination with thermal excitation, show a distinctly higher intensity for the band previously assigned to the overtone of *ν*_9_ (1305 cm^–1^ (ref. 37)) compared to *ν*_5_ (1223 cm^–1^ (ref. 37)), making this a fascinating disagreement of IR and Raman intensity patterns to be resolved.^8^,^45^ Indeed, a very recent IR investigation^55^ points into the same direction.

## Conflicts of interest

There are no conflicts to declare.
